# In Vitro Effects of Pumpkin (*Cucurbita moschata*) Seed Extracts on *Echinococcus granulosus* Protoscoleces

**Published:** 2020

**Authors:** Zahra HESARI, Meysam SHARIFDINI, Mohammad Kazem SHARIFI-YAZDI, Saeedeh GHAFARI, Sara GHASEMI, Shahram MAHMOUDI, Mehdi MOHEBALI, Bahram NIKMANESH

**Affiliations:** 1. Department of Pharmaceutics, School of Pharmacy, Guilan University of Medical Sciences, Rasht, Iran; 2. Department of Medical Parasitology and Mycology, School of Medicine, Guilan University of Medical Sciences, Rasht, Iran; 3. Zoonosis Research Center, Tehran University of Medical Sciences, Tehran, Iran; 4. Department of Medical Laboratory Sciences, School of Allied Medical Sciences, Tehran University of Medical Sciences, Tehran, Iran; 5. School of Traditional Medicine, Shahid Beheshti University of Medical Sciences, Tehran, Iran; 6. Department of Parasitology and Mycology, School of Public Health, Tehran University of Medical Sciences, Tehran, Iran

**Keywords:** *Cucurbita moschata*, Pumpkin seed extract, *Echinococcus granulosus*, Scolicidal effect

## Abstract

**Background:**

*Echinococcus granulosus* parasite causes a zoonotic disease which is important for public and veterinary health. Since pumpkin seeds (*Cucurbita* sp.) are used as traditional vermifuge in Iran, they may be a potential herbal anthelmintic.

**Methods:**

This study was designed in 2016 to evaluate the in vitro scolicidal effect of *Cucurbita moschata* seeds form northern part of Iran. Hydroalcoholic and petroleum ether extracts were prepared by maceration and soxhlet respectively. Both extracts with four different concentrations (100, 10, 1, 0.1 mg/ml) were incubated against protoscoleces in 5, 15, 30 and 60 min.

**Results:**

Maximum mortality was 16% with 1% hydroalcoholic extract in 60 min. The highest mortality with organic extract was 4% with 10% concentration in 60 min (*P*=0.015).

**Conclusion:**

Since highest mortality was 16%, the extract did not reach to LD50 (50% mortality). Therefore, the potency of the total extract is not sufficient as potential scolicidal drug.

## Introduction

Human hydatid cyst is a somehow neglected zoonotic parasitic disease caused by *Echinococcus granulosus* parasite (dog tapeworm) in its larval stage (1). Parasitic infections impair animal health, welfare, and productivity since the presence of worms results in increased death rate and poor growth and reproduction (2). This chronic infection is in importance according to public health and veterinary issues in endemic areas such as Iran (3–5). Even in non-endemic countries, the potential of new endemicity creation, by livestock importations and migration of infected patients, should be considered (6).

At present, there are four main options for cyst treatment; surgery, drug therapy, PAIR (puncture, aspiration, injection, and reaspiration) and observation for inactive cysts; which one or two will be incorporated depending on cyst characteristics and physician’s choice (7). Surgery is the first suggested treating option due to potential of total removal of the cyst and obtaining a 100% cure. However, in the case of inoperable patients or for prevention of cyst recurrence and secondary infection as a result of protoscoleces leakage or even as injection solution after aspiration of cyst contents, incorporation of efficacious scolicidal agents seems to be necessary (8).

The mainly used chemotherapeutics for metacestode stage of *Echinococcus* are benzimidazole carbamates, such as albendazole and mebendazole, in addition to praziquantel and amphotericin B. Unfortunately, a notable number of patients do not respond desirably to these drugs and the rate of relapse after treatment cessation is high. Moreover, adverse drug reactions have been reported because of chemotherapy including liver enzymes elevation, gastrointestinal discomforts, alopecia etc. by albendazole. Similarly, numerous chemical scolicidal solutions have been employed for inactivation of protoscoleces in PAIR and surgery including ethanol, hypertonic saline, silver nitrate, cetrimide, povidone-iodine, albendazole sulfoxide, octenidine hydrochloride and chlorhexidine gluconate; but the majority of them are along with hepatobiliary adverse reactions. Although there is no means by use of scolicidal agents, an ideal one that would be inexpensive, with rapid and complete scolicidal action and leaving minimum local and systemic side effects, has not been discovered yet (9).

A recently developing field with an ancient historical foundation is the use of plant-derived compounds depending on their anthelmintic potentials, in order to discover the ideal scolicidal agent. Plant based medicine mostly favors the advantages of high availability, low cost and probably fewer side effects (10).

Amongst the plants offering Anthelmintic properties, pumpkin seed (*Cucurbita* sp.) is prominent (11). *C. moschata* and *C. maxima* are the main species in Cucurbitaceae used as traditional vermifuge in Iran. Pumpkin is also suggested for treatment of malaria, cough producing chest pain, thirst-quenching and in the form of oil for earache and as gastrointestinal laxative and emollient in traditional Persian medicine (TPM) (12). Due to the vast pumpkin’s therapeutic indications in TPM and appropriate agricultural climate, Iran has been identified among the major pumpkin producing countries by The Food and Agriculture Organization of the United Nations (FAOSTAT) (13).

Effectiveness of pumpkin seeds against taeniasis (11) and schistosomiasis (14) which both are members of platyhelminths phylum, has been previously reported in ethnoveterinary literature and human studies. Moreover, the in vitro anthelmintic activity of pumpkin seed’s extract on *Haemonchus contortus* is confirmed (15).

Based on TPM and recent vermifugal observations of pumpkin seed, this study was designed to evaluate the anthelminthic activity of *C. moschata* spices, which is mainly agriculture and consumed in Iran, against protoscoleces of hydatid cyst. For coverage of a range of polar and nonpolar possible vermicide compounds, both hydro-alcoholic extract (HAE) and petroleum ether extract (PEE) of pumpkin seed was prepared and evaluated.

## Materials and Methods

### Collection and extraction of plant

Pumpkin (*C. moschata*) fruits were bought from local people in northern part of Iran in Aug 2016. Pumpkin seeds were collected from the fruits with the primary weight of about 290 g after washing. Seeds were subjected to dry, in oven in 45 °C until their weight get fixed at 231.43 gr. Afterward, two extraction systems were conducted on 100 g of grounded seeds including hydro-alcoholic extraction for polar compounds and petroleum ether extraction for non-polar compounds.

First grounded seeds were loaded on filter paper and placed in a soxhlet extractor. Plant part was covered by 650 ml petroleum ether solvent and extraction was performed at 65 °C for 8 h (until the solvent appears colorless and clear in the upper part). Then the obtained extract was filtered and solvent amount was decreased to the possible minimum level, utilizing rotary evaporator with vacuum. Remaining plant residue was dried for hydro-alcoholic extraction.

Hydro-alcoholic extract was prepared by maceration method; dried seed powder was mixed with 300 ml hydro-alcoholic solvent (methanol 70: water 30) in an Erlenmeyer flask. Flask was covered with an aluminum foil and shaked overnight. The extract was filtered and kept at 4 °C. Maceration was repeated three times. Methanol part of total extract was evaporated by rotary evaporator. Remaining water part was subjected to freeze drier to make concentrated powder extract. All the extracts were stored in 4 °C in amber bottles.

### Extract dilutions

Both PEE and HAE were serially diluted in distilled water to reach the concentrations of 200, 20, 2 and 0.2 mg/ml. Extracts would be double diluted while mixing with equal volume of protoscolex suspension and therefore present their possible vermicidal effect with final concentrations of 100, 10, 1 and 0.1 mg/ml. Due to hydrophobic nature of petroleum ether extract, tween 80 was used to form a homogenous emulsion with concentration of 10% V/V in extracts and control solutions.

### Anti-helminthic evaluations

#### Collection of protoscoleces

*E. granulosus* protoscoleces were collected from sheep livers slaughtered at Rasht industrial abattoir. Hydatid fluid was aspirated from a cyst by a sterile 50 ml syringe and transferred into a falcon tube. Gentle centrifugation was applied on hydatid fluid with 1000 rpm for 1 min to settle down the protoscoleces with minimum time waste. Protoscoleces were washed with NaCl 0.9% solution and their number was adjusted to 2500 protoscoleces per ml with at least 90% primary viability. Viability was determined by impermeability to eosin dye (Sigma-aldrich) solution under light microscope (Carl-Zeiss Standard 14).

### Effects of pumpkin seed extracts on protoscoleces

#### Viability assessment

For evaluation and comparison of scolicidal activity of HAE and PEE of pumpkin seeds, four different concentrations (100, 10, 1, 0.1 mg/ml) were tested against protoscoleces in 5, 15, 30 and 60 min. Normal saline 0.9% solution was incorporated as control. First 500 μlit of protoscolex suspension was loaded in 1.5 ml micro-tubes, then 500 μlit of each concentration was added to micro-tubes. Tube contents were gently mixed and incubated in 37 °C for 15, 30 and 60 min. In each time point, 50 μlit of settled protoscoleces was mixed with 50 μlit of eosin dye 0.1% on a glass slide and covered with a cover glass. The slide was carefully studied under a light microscope and the percentage of dead protoscoleces was calculated after total counting of 300 protoscoleces. After exposure to protoscolex with eosin dye, dead organisms absorbed the dye and appeared with orange-red color. While live protoscoleces remained impermeable to eosin (colorless) and maintained their flame cell activity and muscular movements. The procedure was separately performed for hydro-alcoholic and petroleum ether extracts and the experiment was run in triplicate for each concentration and time interval.

#### HPTLC Analysis of pumpkin seed extracts

**Solvent extractions**. 0.1 g of extracts was subjected to serial extraction with four organic solvents, covering a wide range of polarity, starting from an absolutely non-polar solvent (hexane) followed by gradually increasing polarity solvents (ethyl acetate, chloroform, methanol). Next, acid and alkaline extraction were performed on methanol components by the use of HCl 0.1M and NH3 10% v/v and final extraction of salts was accomplished with chloroform.

**Instruments.** Silica gel 60 F254 glass plates (10 × 20 cm with 200 μm thickness HPTLC; Merck), CAMAG automatic TLC Samoler 4 (ATS 4), CAMAG automatic Developing Chamber 2 (ADC 2), CAMAG TLC Visualizer and winCATS version 1.4.4 software (CAMAG) were used in this study.

**Chromatographic experiment.** Sample solutions, 10 μL each, were applied on the TLC plate using (ATS 4) in the form of band (bandwidth: 6 mm, distance between two bands: 9.4 mm). A constant application rate of 150 nl s-1 was used with the mobile phase of chloroform-methanol (8:2) v/v. The plates were then placed in the mobile phase, and ascending development was performed to a distance of 7 cm. Subsequent to the development, the plates were air-dried and Chromatogram evaluated with TLC Visualizer under 254 nm, under 366nm and white light.

**Phytochemical Screening.** Thin layer chromatographic (TLC) analysis was performed qualitatively on hydro-alcoholic extract of *C. moschata* seed. The mobile phase system was chloroform-methanol (7:3) and silica gel 60 F 254 HPTLC plate was incorporated as stationary phase. Five different reagent including anisaldehyde-sulfuric acid, dragendorff‘s, ninhydrin, potassium hydroxide, sulfuric acid; were utilized to describe secondary metabolic compounds found in seed’s extracts.

### Statistical analysis

All statistical analysis was performed using t-test with SPSS 18.0 (Chicago, IL, USA). Statistically significant differences between control and samples were confirmed by *P*-value <0.05. All of experiments were repeated three times (n=3).

## Results

### Plant Extraction

Total weight of petroleum ether and hydro-alcoholic extracts were 23.43 g and 2.49 g respectively.

### In vitro effect on protoscoleces

**Fig. 1:**
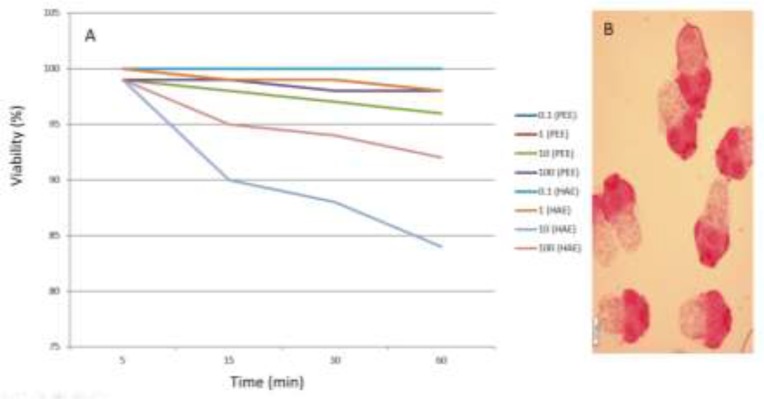
A) Viability percent of *E. granulosus* protoscoleces incubated with different concentrations of Hydroalcoholic extract (HAE) as compared with the same concentrations of petroleum ether extract (PEE). B) Dead protoscolices of *E. granulosus* after exposure to pumpkin seed extract with 0.1% eosin

The survival of *E. granulosus* protoscoleces incubated with different concentrations of HAE as compared with the same concentrations of PEE is shown in Fig. 1.

The mean mortality increased from 0% to 4% in 5 to 60 min respectively in concentration of 10 mg/ml of PEE. Moreover, the mean mortality grows from 0% to 16% among the increasing concentration of HAE from 0.1 to 100 mg/ml. PEE showed dose and time-dependent scolicidal activities, except concentration of 100 mg/ml, which showed lower scolicidal effect than the concentration of 10 mg/ml Table 1. Furthermore, HAE produced only a time-dependent activity but not repeating the same manner in dose dependency. Mean mortality increased from 0% to 16% in 5 to 60 min respectively in concentration of 1mg/ml. On the other hand, the highest scolicidal effect was obtained by concentration of 1 mg/ml and then 10, 100, 0.1 mg/ml respectively Table 2.

**Table 1:** % viability of protoscoleces while incubation with petroleum ether extract (PEE) of pumpkin seed

***Extract concentration***	***Protoscoleces viability (%) Mean ±SD***			

(mg/ml)	5 mins	15 mins	30 mins	60 mins
0.1	100 (0.0)	100 (0.0)	100 (0.0)	100 (0.0)
1	100(0.0)	99 (1.0)	99 (1.0)	98 (1.0)
10	99 (1.0)	98 (1.0)	97(1.0)	96(1.0)
100	99(1.0)	99(1.0)	98 (1.0)	98 (1.0)

**Table 2:** Percentage of viability of protoscoleces while incubation with hydro-alcoholic extract (HAE) of pumpkin seed

***Extract concentration (mg/ml)***	***Protoscoleces viability (%) Mean ±SD***			

	5 mins	15 mins	30 mins	60 mins
0.1	100 (0.0)	100 (0.0)	100 (0.0)	100 (0.0)
1	100(0.0)	99 (1.0)	99 (1.0)	98 (1.0)
10	99 (1.0)	90 (5.0)	88 (2.0)	84 (1.0)
100	99(1.0)	95 (2.0)	94 (4.0)	92 (2.0)

### Phytochemistry and HPTLC analysis

**Fig. 2:**
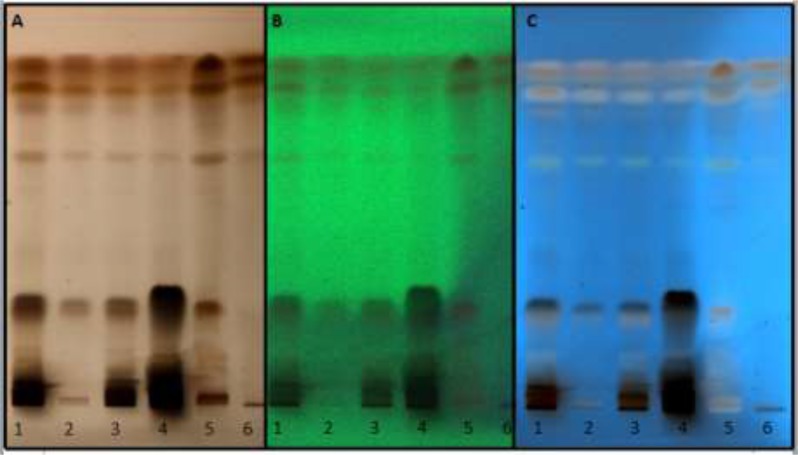
HPTLC analysis of HAE before and after derivatization under A) white light and UV light B) 366 nm and C) 254 nm. HAE was again extracted with: 1-Hexane 2-Ethyl acetate 3-chloroform 4-methanol 5-HCL 6-NH3

**Fig. 3:**
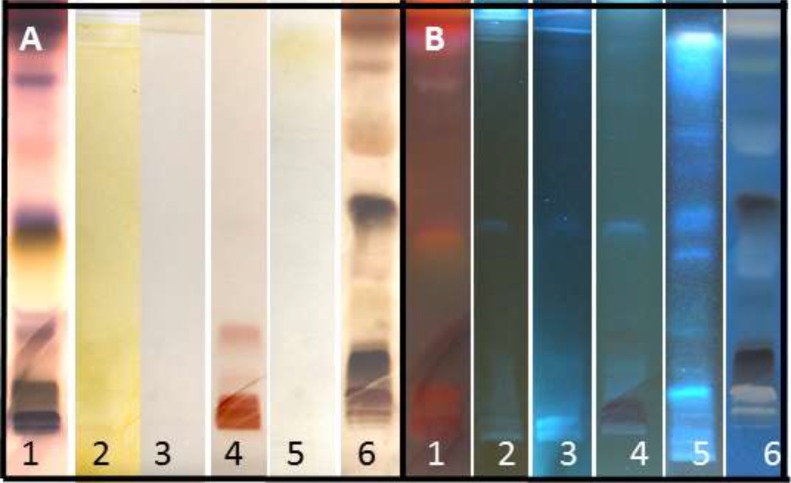
phytochemical analysis of HAE under A) visible and B) UV light with: 1- Anisaldehydesulfuric Acid 2- Dragendorff‘s 3- Natural products 4- Ninhydrin 5- Potassium hydroxide 6- Sulfuric acid

Due to higher scolicidal effect of HAE, HPTLC analysis was performed to investigate its components. Analysis of samples before and after derivatization under white light and UV light (366 and 254 nm) was presented in Fig. 2. Preliminary phytochemical analysis of hydroalcoholic extract of pumpkin seed revealed the presence of terpenoids, saponins, esterols and amino acids based on color zones obtained Fig. 3.

## Discussion

Following WHO declaration about the necessity of discovering or developing an ideal inexpensive agent, with rapid and complete scolicidal action with minimum local and systemic side effects (16), various studies have focused on in vitro/vivo efficacy of several herbal extracts/oils against *E. granulosus* protoscoleces in order to detect a promising source of a new protoscolicidal component.

To the best of our knowledge, despite many other investigations on anti-microbial activities of pumpkin seed, it is the first time that the anti-helminthic effect against protoscoleces is evaluated. *C. moschata* seed has an in vitro scolicidal effect, which is stronger in HAE in comparison with PEE. Paying attention to highest mortality rate that in PEE, was reached to 4% with concentration of 10 mg/ml in 60 min and in HAE was 16% in concentration of 1 mg/ml in 60 min it was revealed that none of the extracts provide sufficient scolicidal activity to reach 50% mortality (LD50) desired for an extract to be candidate for use as a drug. However, in vivo effect of pumpkin seed or its components was confirmed on ostrich nematodes (17), mice schistosomiasis (18), and canine tapeworms (19). Moreover, combination of pumpkin seed and areca nut effectiveness was investigated in heterophyiasis in puppies (20) and human taeniasis (11).

On the other hand, the in vitro anthelmintic effect of *C. moschata* seed extract was confirmed on Haemonchus contortus (15). Phytochemical studies affirm presence of various constituents in pumpkin seed including terpenoids, amino acids and saponins. Of effective components are Cucurbitacins chemically classified as steroids and function as a defense against herbivores. Marie-Magdeleine et al. discovered cucurbitacin in pumpkin seed’s water and methanol extract with ninhydrin reagent also confirmed in our hydroalcoholic extract as brown spot in reaction with ninhydrin (15). Pumpkins, collected from Guadeloupe (French West Indies) (15) revealed similar constituents as pumpkins collected from northern Iran.

## Conclusion

However, the *C. moschata* extracts (especially hydroalcoholic) showed scolicidal activity, since highest mortality was 16% with concentration of 10 mg/ml of HAE in 60 min, the extract did not reach LD50 (50% mortality). Therefore, the potency of the total extract is not sufficient as potential scolicidal drug. Moreover, further studies on other solvents and extraction methods and phytochemical analysis in order to find the exact efficacious molecule can be impressive.
